# Evolution of the Relaxin/Insulin-Like Gene Family in Anthropoid Primates

**DOI:** 10.1093/gbe/evu023

**Published:** 2014-02-02

**Authors:** José Ignacio Arroyo, Federico G. Hoffmann, Juan C. Opazo

**Affiliations:** ^1^Instituto de Ciencias Ambientales y Evolutivas, Facultad de Ciencias, Universidad Austral de Chile, Valdivia, Chile; ^2^Programa de Doctorado en Ciencias mención Ecología y Evolución, Facultad de Ciencias, Universidad Austral de Chile; ^3^Department of Biochemistry, Molecular Biology, Entomology and Plant Pathology, Mississippi State University; ^4^Institute for Genomics, Biocomputing and Biotechnology, Mississippi State University

**Keywords:** relaxin, differential gene retention, convergent evolution, gene duplication, positive selection

## Abstract

The relaxin/insulin-like gene family includes signaling molecules that perform a variety of physiological roles mostly related to reproduction and neuroendocrine regulation. Several previous studies have focused on the evolutionary history of relaxin genes in anthropoid primates, with particular attention on resolving the duplication history of *RLN1* and *RLN2* genes, which are found as duplicates only in apes. These studies have revealed that the *RLN1* and *RLN2* paralogs in apes have a more complex history than their phyletic distribution would suggest. In this regard, alternative scenarios have been proposed to explain the timing of duplication, and the history of gene gain and loss along the organismal tree. In this article, we revisit the question and specifically reconstruct phylogenies based on coding and noncoding sequence in anthropoid primates to readdress the timing of the duplication event giving rise to *RLN1* and *RLN2* in apes. Results from our phylogenetic analyses based on noncoding sequence revealed that the duplication event that gave rise to the *RLN1* and *RLN2* occurred in the last common ancestor of catarrhine primates, between ∼44.2 and 29.6 Ma, and not in the last common ancestor of apes or anthropoids, as previously suggested. Comparative analyses based on coding and noncoding sequence suggests an event of convergent evolution at the sequence level between co-ortholog genes, the single-copy RLN gene found in New World monkeys and the *RLN1* gene of apes, where changes in a fraction of the convergent sites appear to be driven by positive selection.

## Introduction

Convergent evolution is defined as the process whereby unrelated organisms independently reach similar character states. At the phenotype level, one of the best known examples of convergence is the wing, in which phylogenetically unrelated groups (e.g., insects, bats, and birds) evolved the ability of flight independently. At the molecular level, several cases have been reported in which preexisting genes have changed their original function (Eizinger et al. 1999; Piatigorski 2007). One remarkable example is the independent evolution of the oxygen-transport hemoglobins between gnathostomes (jawed vertebrates) and cyclostomes (jawless vertebrates) (Hoffmann et al. 2010). An important issue regarding convergent evolution is to understand the role of different evolutionary forces that are behind the process to understand the mechanisms of functional adaptation. Although convergent evolution represents an important mechanism to promote evolutionary innovations, detecting convergent events represents a challenge especially when the duplicative history of the genes is complex, and orthologous relationships are not well understood.

The relaxin/insulin-like gene family includes signaling molecules that perform a variety of physiological roles mostly related to reproduction and neuroendocrine regulation (Bathgate et al. 2003; Sherwood 2004; Park et al. 2005; McGowan et al. 2008). Recent analyses revealed that the two whole genome duplications that occurred early in vertebrate evolution are linked to the initial expansion of this group of genes (Hoffmann and Opazo 2011; Yegorov and Good 2012). Members of this gene family are found on three different genomic locations in mammals, which have been called relaxin family locus (RFL) A, B, and C (Park et al. 2008).

**Fig. 1.—:**
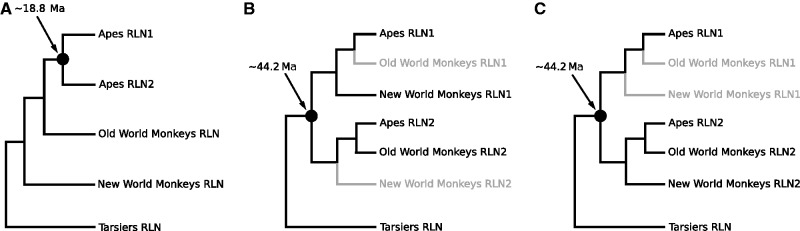
Schematic representations of alternative hypotheses regarding phylogenetic relationships among the duplicated RLN genes in anthropoid primates. In (*A*) RLN1 and RLN2 genes arose via duplication of a proto-RLN gene in the last common ancestor of apes. In (*B*) the duplication event that gave rise to RLN1 and RLN2 genes predates the radiation of anthropoid primates, although a two gene arrangement was present in the last common ancestor of anthropoid primates, only apes appear to have retained both copies, whereas New and Old World monkeys independently retain complementary gene copies, RLN1 and RLN2, respectively. In (*C*), the duplication event also predates the radiation of anthropoid primates but this time New and Old World monkeys have independently retained the RLN2 paralog. Lineages in gray denote gene losses.

The number and nature of genes in these three genomic loci are well conserved in most mammalian lineages, with the exception of the RFLB locus (Park et al. 2008; Hoffmann and Opazo 2011; Arroyo, Hoffmann, Good, et al. 2012; Arroyo, Hoffmann, Opazo 2012a, 2012b). This locus possess a complex duplicative history characterized by small-scale duplications and differential gene retention, where the relative age of many genes is not consistent with their phyletic distribution (Hoffmann and Opazo 2011; Arroyo, Hoffmann, Good, et al. 2012; Arroyo, Hoffmann, Opazo 2012a, 2012b). For example, the INSL4 gene, also called placentin, is restricted to catarrhine primates but derives from a duplication event in the last common ancestor of placental mammals (Bieche et al. 2003; Park et al. 2008; Park, Semyonov, et al. 2008; Arroyo, Hoffmann, Good, et al. 2012; Arroyo, Hoffmann, Opazo 2012b). This is also true for the RLN1 and RLN2 paralogs of anthropoid primates (Wilkinson et al. 2005; Park et al. 2008; Park, Semyonov, et al. 2008; Hoffmann and Opazo 2011; Arroyo, Hoffmann, Good, et al. 2012; Arroyo, Hoffmann, Opazo 2012b), for which multiple competing scenarios have been proposed to explain their evolutionary origin. Initial studies postulated that the duplication event that gave rise to the RLN1 and RLN2 genes, which are only found in duplicate in apes, occurred in their last common ancestor (fig. 1*A*; Evans et al. 1994; Wilkinson et al. 2005; Park et al. 2008; Park, Semyonov, et al. 2008; Hoffmann and Opazo 2011). In this scenario, the RLN1 and RLN2 genes in apes would be co-orthologs to the single copy RLN gene found in most mammals. More recently, Arroyo, Hoffmann, Opazo (2012b) suggested that RLN1 and RLN2 originated in the last common ancestor of anthropoid primates, and were only retained as duplicates in apes, whereas New and Old World monkeys independently lost copies of RLN1 and RLN2, respectively (fig. 1*B*). Here, the single copy RLN gene from New World monkeys would be a 1:1 ortholog to the RLN1 gene of apes, whereas the single copy RLN gene from Old World monkeys would be a 1:1 ortholog to the RLN2 gene of apes. However, dot-plot comparisons suggested the possibility that the RLN gene found in New World monkeys could be a 1:1 ortholog to the RLN2 gene of apes (fig. 1*C*; Arroyo, Hoffmann, Opazo [2012b]). Thus, the relationships among these genes remained unresolved.

The main goal of this research is to unravel the history of duplication of the RLN1 and RLN2 genes of anthropoid primates to estimate the timing of the duplication that gave rise to the RLN1 and RLN2 genes, and asses the potential role of natural selection in their divergence. To this end, we contrasted phylogenies based and coding and noncoding sequences, and compared rates of synonymous and nonsynonymous substitution along the tree based on coding sequences. Results from our phylogenetic analyses based on noncoding sequence revealed that the duplication event that gave rise to the RLN1 and RLN2 genes occurred in the last common ancestor of catarrhine primates, between ∼44.2 and 29.6 Ma, and not in the last common ancestor of apes or anthropoids, as previously inferred. Comparative analyses based on coding and noncoding sequence suggest an event of convergent evolution at the sequence level between co-ortholog genes, the single-copy RLN gene found in New World monkeys and the RLN1 gene of apes. Molecular evolution analyses suggest that changes in some of the convergent sites appear to be driven by positive selection, and also suggest that the peptide C from the relaxin precursor might play functionally relevant roles that need to be explored

## Materials and Methods

### DNA Sequence Data

We manually identified relaxin/insulin-like genes that belong to the Relaxin Family Locus B (RFLB) in 15 species of primates representing all main groups of the order (supplementary table S1, Supplementary Material online). The primates species included six apes (human, *Homo sapiens*; chimpanzee, *Pan troglodytes*; bonobo, *P. paniscus*; gorilla, *Gorilla gorilla*; orangutan, *Pongo abelii*, and gibbon, *Nomascus leucogenys*), four Old World monkeys (rhesus macaque, *Macaca mulatta*; crab-eating macaque, *M. fascicularis*; olive baboon, *Papio anubis;* and hamadryas baboon, *Pap. hamadryas*), two New Wold monkeys (squirrel monkey, *Saimiri boliviensis* and marmoset, *Callithrix jacchus*), one tarsier (*Tarsius syrichta*), and two strepsirrhines (mouse lemur *Microcebus murinus*, and bushbaby, *Otolemur garnetti*). We compared annotated exons sequences with unannotated genomic sequences using the program Blast2seq (Tatusova and Madden 1999). Putatively functional genes were characterized by an intact open reading frame with the canonical two exon/one intron structure typical of vertebrate RLN/INSL-like genes, whereas pseudogenes were identifiable because of their high sequence similarity to functional orthologs and the presence of inactivating mutations, and/or the lack of exons. To distinguish among tandemly arrayed genes copies, we index each gene copy with the symbol T followed by a number that corresponds to the linkage order in the 5 to 3′ orientation, thus, the first gene in the cluster is labeled T1, the second T2, and so forth. Pseudogenes were indexed with the ps suffix.

### Phylogenetic Inference

We estimated phylogenetic relationships among RLN genes in all major groups of primates. We used a maximum likelihood and a Bayesian analyses, as implemented in the programs Treefinder version March 2011 (Jobb et al. 2004) and Mr.Bayes v3.1.2 (Ronquist and Huelsenbeck 2003), respectively. Because convergent evolution is typically restricted to the coding regions, in addition to using phylogenetic reconstructions based on coding sequence, we also used noncoding sequences (flanking regions and intron 1) to unravel the evolutionary history of the RLN genes in anthropoid primates. Sequence alignments were carried out using the L-INS-i strategy from MAFFT v.6 (Katoh et al. 2009). In the case of the coding sequence, the best fitting models for each structural domain (signal peptide, and peptides B, C, and A) was estimated separately using the propose model routine from the program Treefinder version March 2011 (Jobb et al. 2004). For noncoding sequences a single model of molecular evolution was estimated for each region (up- and downstream flanking sequences, and intron 1). In the case of maximum likelihood, we estimated the best tree under the selected models, and assessed support for the nodes with 1,000 bootstrap pseudoreplicates. In Bayesian analysis, two simultaneous independent runs were performed for 10 × 10^6^ iterations of a Markov Chain Monte Carlo algorithm, with six simultaneous chains sampling trees every 1,000 generations. Support for the nodes and parameter estimates were derived from a majority rule consensus of the last 5,000 trees sampled after convergence. The average standard deviation of split frequencies remained 0.01 after the burn-in threshold.

### Molecular Evolution Analysis

To investigate the possible role of natural selection in the evolutionary history of the RLN gene of New World monkeys, we explored variation in ω, the ratio of the rate of nonsynonymous and synonymous substitutions per nonsynonymous and synonymous site, in a maximum likelihood framework using the program *codeml* from the PAML v4.4 package (Yang 2007). We compared two sets of models, the first set focused on comparing changes in ω ( = *d*_N_/*d*_S_) along the branches of the tree, and the second set of models focused on comparing changes in ω along the different sites in the alignment between background and foreground sets of branches. We first compared the following two branch models: 1) a 1 − ω model in which a single ω estimate was assigned to all branches in the tree; and 2) a 2 − ω model, which assigned one ω to the ancestral branch of the New World monkey RLN clade, and a second ω to all other branches. We also implemented branch-site models, which explore changes in ω for a set of sites in a specific branch of the tree to assess changes in their selective regime (Yang and dos Reis 2011). In this case, the ancestral branch of the New World monkey RLN clade was labeled as the foreground branch. We compared the modified model A (Yang et al. 2005; Zhang et al. 2005), in which some sites are allowed to change to an ω > 1 in the foreground branch, with the corresponding null hypothesis of neutral evolution. The Bayes Empirical Bayes (BEB) method was used to identify sites under positive selection (Nielsen and Yang 1998; Yang et al. 2000). Because the branch-site analysis estimates rates of evolution on a codon by codon basis, its implementation is particularly useful in cases when different gene segments evolve at different rates, as is the case with the different domains of the RLN genes.

## Results and Discussion

The evolutionary history of the relaxin genes in anthropoid primates has been intensely studied (Evans et al. 1994; Wilkinson et al. 2005; Park et al. 2008; Park, Semyonov, et al. 2008; Hoffmann and Opazo 2011; Arroyo, Hoffmann, Opazo 2012b). Most studies have focused on resolving the duplicative history of the RLN1 and RLN2 genes of apes. These studies suggest that the RLN1 and RLN2 paralogs of apes have a more complex history than their phyletic distribution suggests. In this regard, three alternative scenarios have been proposed to explain the timing of duplication and gene gains and losses along the organismal tree (fig. 1*A*–*C*). Initial studies had suggested that the duplication giving rise to RLN1 and RLN2 mapped to the last common ancestor of apes, between approximately 29.6 and 18.8 Ma (fig. 1*A*; Evans et al. 1994; Wilkinson et al. 2005; Park et al. 2008; Park, Semyonov, et al. 2008), but phylogenies with more extensive taxonomic sampling suggested that the same duplication mapped to the last common ancestor of anthropoid primates, the group that includes apes and Old and New World monkeys, between ∼71.1 and 44.2 Ma. The identity of the RLN gene lost by New and Old World monkeys remained unclear (fig 1*B* and *C*; Arroyo, Hoffmann, Opazo 2012), as support for the relevant nodes was not significant to resolve among competing alternatives.

The phylogenetic evidence presented by Arroyo, Hoffmann, Opazo (2012b) suggested an older origin than previously proposed, but it was not conclusive (Wilkinson et al. 2005; Park et al. 2008; Park, Semyonov, et al. 2008; Hoffmann and Opazo 2011). Phylogenetic analyses of paralogous members of a gene family often result in nonorthologous genes appearing more similar to each other than they are to their true orthologs. In particular, gene conversion and positive Darwinian selection often obscure phylogenetic reconstructions among paralog members of a gene family. However, because both gene conversion and positive Darwinian selection are largely restricted to coding regions, true homologous relationships can often be determined by analyzing variation in introns and flanking sequence. Accordingly, we expanded our phylogenetic analyses of the RLN1 and RLN2 paralogs of primates to include noncoding sequences corresponding to the single intron plus the upstream and downstream flanking regions, and also explored the role of natural selection in the evolution of the coding sequence of these genes.

**Fig. 2.—:**
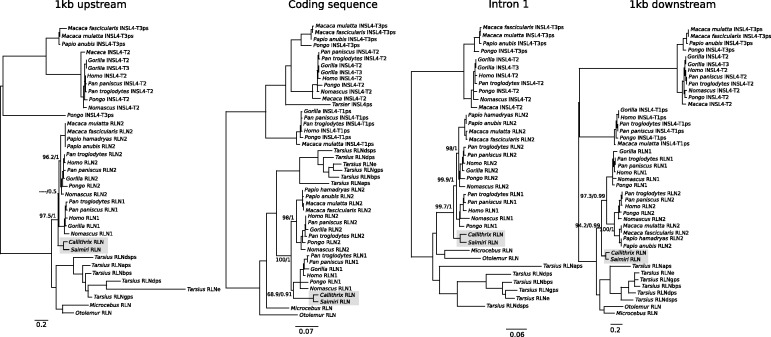
Maximum likelihood phylograms depicting relationships among relaxin-like genes in primates based on 1 kb of 5′ flanking sequence, coding sequence, intron 1, and 1 kb of 3′ flanking sequence. Numbers on the nodes correspond to maximum likelihood bootstrap support values and Bayesian posterior probabilities. Single copy RLN gene found in New World monkeys are shaded.

**Fig. 3.—:**
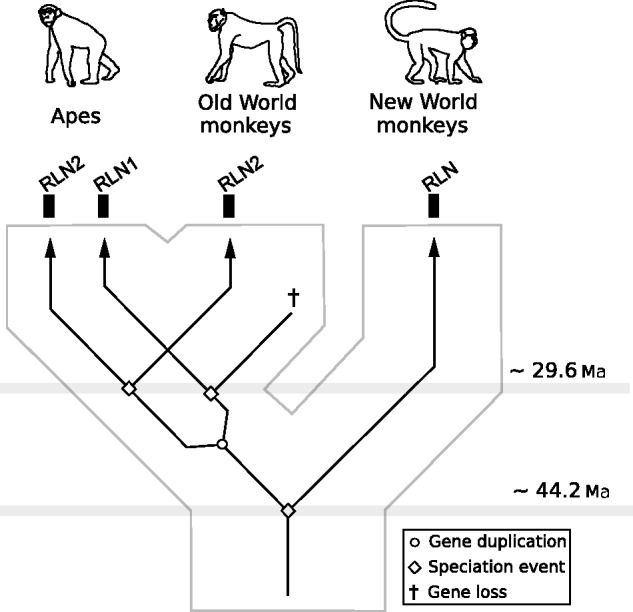
An evolutionary model for the evolution of the RLN1 and RLN2 genes in anthropoid primates. The model indicates that the RLN1 and RLN2 paralogs derive from the duplication of a proto-RLN gene in the last common ancestor of catarrhine primates, and not in the last common ancestor of apes or anthropoids as previously thought. Although a two gene arrangement was present in the last common ancestor of catarrhine primates, only apes appear to have retained both copies, whereas Old World monkeys lost the RLN1 paralog.

In all analyses the two RLN1 and RLN2 paralogs of apes fell in two separate clades that did not deviate significantly from the expected organismal phylogenies (fig. 2). Thus, we infer that these phylogenies resolved orthology among the RLN1 and RLN2 paralogs of apes, with the exception of a small conversion tract in the first exon restricted to chimps and bonobos (Evans et al. 1994). Interestingly, phylogenies based on coding and noncoding sequences gave contrasting answers regarding the position of the single copy RLN gene of New World monkeys (fig. 2). As in Arroyo, Hoffmann, Opazo (2012b), phylogenies based on coding sequence placed the single copy RLN gene of New World monkeys as sister to the RLN1 genes of apes (fig. 2). A tree topology suggesting that the duplication that gave rise to the RLN1/RLN2 paralogs occurred in the last common ancestor of anthropoid primates (Arroyo, Hoffmann, Opazo 2012b). However, phylogenies based on the three separate noncoding fragments consistently placed the New World monkey RLN genes as sister to the clade containing RLN1/RLN2 sequences from Old World monkeys and apes (fig. 2). This result would suggest a novel alternative to the three evolutionary scenarios already proposed in which the RLN1 and RLN2 paralogs would derive from the duplication of a proto-RLN gene in the last common ancestor of catarrhine primates, between ∼44.2 and 29.6 Ma (fig. 3). According to this novel scenario, the single copy RLN gene of New World monkeys represents the ancestral condition, whereas the single copy RLN gene of Old World monkeys would derive from the secondary loss of the RLN1 paralog in the group (fig. 3). This was also supported by approximately unbiased topology tests (Shimodaira and Hasegawa 1999), based on the intron or downstream alignments, which rejected the placement of the New World monkeys RLN gene as sister to the RLN1 gene of apes (*P* < 0.001). Because the observed differences between coding and noncoding phylogenies were statistically significant, our results are indicative of a pattern of convergent evolution at the sequence level.

Phylogenetic reconstructions have been widely used in the literature to investigate events of putative convergent evolution at the sequence level (Castoe et al. 2009; Li et al. 2010; Liu et al. 2010; Yokoyama et al. 2011). Cases where species with similar phenotypes are grouped together rather than with their true relatives have been considered as evidence for convergent evolution, defined here in a loose manner to include both convergent and parallel evolution. For example, Liu et al. (2010) studied the evolution of prestin genes, which encode for a protein involved in hearing, and found that a process of convergent evolution driven by natural selection was responsible for the placement of the dolphin gene within a clade that included echolocating microbats rather than to the cow, which was its true closest relative.

In this case, we investigated the potential role of natural selection on the evolution of the single copy RLN gene of New World monkeys. In particular, we focused on exploring the possibility that the phylogenetic affinity between the RLN gene from New World monkeys and the RLN1 paralog of apes are due to convergent evolution at the sequence level driven by natural selection. If this was the case, we hypothesized that the branch leading to the RLN gene of New World monkeys would have a *d*_N_*/d*_S_ ratio significantly higher than 1, and that some of the codons under natural selection could have converged to the same state independently in both lineages.

To test the first of these predictions, we explored variation in ω ( = *d*_N_*/d*_S_) among the branches in the tree in a maximum likelihood framework. First, we compared a 2 − ω model that assigned one independent ω estimate with the ancestral branch of the RLN clade of New World monkeys and a second one to the rest of the tree with a 1 − ω model where all branches were assigned the same ω*.* The 2 − ω model was significantly better according to the likelihood ratio test (LRT = 6.32, *P* < 0.02). Under the 2 − ω model, the ancestral branch of the New World monkey RLN clade had an ω estimate of 1.77 whereas all other branches had an ω of 0.76 (table 1). The branch-site analyses yielded similar results, as the LRTs favored the alternative model (LRT = 3.86, *P* = 0.049), where several residues switched to a positive selection regime in the ancestral branch of the New World monkeys RLN clade. The BEB analysis identified 35 codons under a positive selection regime, two on the region encoding for the signal peptide, four on the region encoding for the B peptide, 21 on the region encoding for the C peptide, and eight located on the region encoding for the A peptide (table 1). These results suggest that positive Darwinian selection in the ancestral branch of the New World monkey RLN clade was responsible for the remodeling of this protein, and probably accounts for the phylogenetic position of the New World monkeys RLN gene in phylogenies derived from coding sequence.

**Table 1:** Log Likelihood and Parameter Estimates under Different Branch and Branch-Site Models

Model	ln *L*	Parameter Estimates	Positively Selected Sites
Branch models			
1 − ω	−4,734.19	ω_all branches_ = 0.799	NA
2 − ω	−4,731.03	ω_non-New World monkey branches_ = 0.758; ω_ancestral branch of the New World monkey RLN clade_ = 1.776	NA
Branch-site models			
ω fixed (NWM)	−4,685.07	*p*_0_ = 0.259; *p*_1_ = 0.347; *p*_2a_ = 0.167; *p*_2b_ = 0.224; ω_0_ = 0.246, ω_1_ = 1; ω_2_ = 1	NA
ω free (NWM)	−4,683.14	*p*_0_ = 0.170; *p*_1_ = 0.228; *p*_2a_ = 0.257; *p*_2b_ = 0.343; ω_0_ = 0.245; ω_1_ = 1; ω_2a/b_ = 3.477	SP: 19, 20; B: 2,4, 22, 28; C: 7, 11, 12, 13, 15, 16, 18, 25, 26, 38, 49, 50, 52, 55, 56, 66, 71, 74, 102, 103, 107; A: 3, 4, 7, 8, 9, 12, 19, 22

Note.—ln *L*, likelihood value; *p,* proportion of site class; ω, omega value for branches or site classes; SP, signal peptide; B, B peptide; C, C peptide C; A, A peptide.

**Fig. 4.—:**
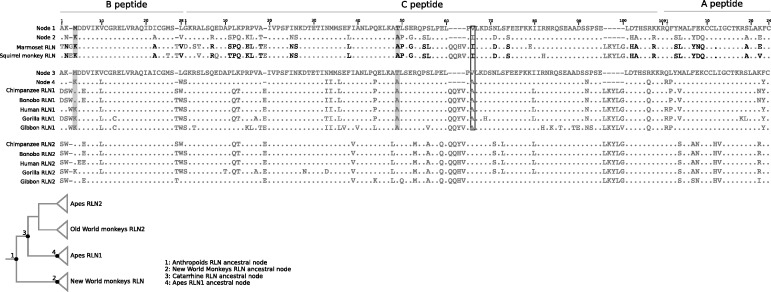
Alignments of relaxin amino acid sequences. The upper panel depicts an alignment of the ancestral states reconstructed for the branch leading to the New World monkey RLN clade (nodes 1 and 2), and two actual New World monkey species. The middle panel shows an alignment of the ancestral states reconstructed for the branch leading to the ape RLN1 clade (nodes 3 and 4), and five actual ape species. The lower panel shows RLN2 sequences from five actual ape species. Amino acids in bold denote sites inferred under positive selection, shaded amino acids are parallel changes, and boxed amino acid is a parallel change where the derived amino acid state was not the same in both lineages but they belong to the same functional group. Amino acid sites labeled with an X were not included in the ancestral sequence reconstruction analysis as the entire columns of gapped sites were removed.

We then explored whether convergence at the nucleotide level resulted in convergence at the amino acid level. In this scenario, a number of the codons under natural selection in the ancestral branch of New World monkey RLN clade would have converged to the same amino acid state as the RLN1 genes of apes. To do so, we reconstructed ancestral sequences of the relevant nodes using a maximum likelihood approach and tracked amino acid changes along the tree (fig. 4). We found that two of the codons inferred to be evolving under positive Darwian selection, B4 and C49, had changed in parallel (fig. 4). In the case of the B4 site, a Met was substituted by a Lys in both ancestral branches, whereas a Thr was substituted by an Ala on the C49 site (fig. 4). We identified one additional positively selected codon, C66, where the derived amino acid state belongs to the same functional group (fig. 4). In this case, a nonpolar/neutral amino acid (ValC66) was replaced by amino acids with the same functional properties (fig. 4). The fact that two amino acid replacements were strictly parallel, and in other case the derived state belongs to the same functional group indicates that a few of the positively selected codons support the convergent hypothesis at the amino acid level. Thus, our analyses would suggest that the sister group relationship between the single copy RLN gene from New World monkeys and the RLN1 paralog of apes is due to an event of convergent evolution at the sequence level between co-ortholog genes, where changes in a subset of the convergent sites appear to be driven by positive selection.

Aside from resolving the evolutionary history of the RLN1 and RLN2 paralogs our results have functional implications as well. Most of the positively selected residues are located on the region encoding for the C peptide, an interesting result given that in marmoset, prorelaxin, the hormone whose C-peptide domain has not been proteolytically cleaved, possesses biological activity similar to the processed peptide (Tan et al. 1998; Zarreh-Hoshyari-Khah et al. 2001; Silvertown et al. 2003). Similar results have been shown for relaxin 3 (Bathgate et al. 2006), suggesting that processing the precursor might not be an essential prerequisite for the acquisition of biological activity. A similar situation has been demonstrated for the proinsulin molecule, a member of a closely related gene family, which is an active agent that binds to the insulin-receptor A, eliciting a differential signaling with enhanced mitogenic effects that regulate embryo development (Hernandez-Sanchez et al. 2006; Malaguarnera et al. 2012). In this regard, proinsulin has been detected in the chick embryo as early as 0.5 days of development, during gastrulation, and also in the retinal neuroepithelium at day 3 (Diaz et al. 1999; Hernandez-Sanchez et al. 2002). In addition to the physiological roles of the C peptide in the unprocessed molecule, it is also involved in the correct folding and disulphide bond pairing of the relaxin molecule. Although its length is approximately 100 amino acids long, it has been shown that the full length is not required to attain the correct molecular conformation (Vandlen et al. 1995). In the particular case of the RLN2 molecule, Vandlen et al. (1995) demonstrated that a C peptide of just 13 amino acids is enough to achieve the correct folding and disulphide bond pairing. Similar results have been shown for the insulin molecule (Busse et al. 1976).

A full exploration of the convergent evolution scenario should be accompanied with physiological data that demonstrates that both proteins, RLN1 from apes and RLN gene from New World monkeys, perform the same physiological function. However, this is difficult to demonstrate at this time, as in a recent review, Bathgate et al. (2013) stated, “The function of the RLN1 gene in humans and higher primates is unknown.” In the same work they also said “The RLN1 gene is only found in humans and the great apes, but in some of these species, it is doubtful that a functional peptide is produced. Even in humans where mRNA expression is detected in multiple tissues, there is no evidence for functional peptide production.” In agreement with these statements, Shabanpoor et al. (2009) wrote, “the mRNA expression of H1 relaxin has been detected in human deciduas, prostate gland and placenta trophoblast. However, its functional significance remains unknown.”

At the expression level it has been reported that the RLN1 gene has a more restricted expression than the RLN2 gene. The RLN1 gene has been detected in the decidua, trophoblast, and prostate (Sakbun et al. 1990; Hansell et al. 1991), whereas the RLN2 gene is expressed in the corpus luteum, endometrium, decidua, placenta, prostate, mammary glands, heart, and brain (Bathgate et al. 2006; Ivell et al. 2011). Accordingly, it could be hypothesized that one of the consequences of a convergent event between the RLN1 of apes and the single copy RLN gene of New World monkeys could be a restriction in the expression pattern of the single copy RLN gene found in New World monkeys. However, given the essential physiological roles of the single copy RLN gene found in the RFLB locus in most mammalian species, we think is highly improbable that in any actual mammal (including NWM) this gene could suffer a restriction on its expression pattern. In support of this claim, it has been shown that in marmoset (*C. jacchus*) the pattern of relaxin expression appears to be very similar to the human (Steinetz et al. 1995; Einspanier et al. 1997, 1999).

## Conclusions

Our results allowed us to refine the current model for the evolution of the RLN1 and RLN2 paralogs in anthropoid primates. According to our phylogenies, the duplication event that gave rise to the RLN1 and RLN2 paralogs occurred in the last common ancestor of catarrhine primates (fig. 3), and not in the last common ancestor of apes or anthropoids, as previously thought. Although both genes were present in the last common ancestor of catarrhine primates, only apes appear to have retained both copies, whereas Old World monkeys lost the RLN1 paralog. This refined model highlights the role of the differential retention of relatively old paralogs in shaping the gene complement in catarrhine primates. In addition, we showed that the sister group relationship between the RLN gene of New World monkeys and the RLN1 paralog of apes was due to convergent evolution at the nucleotide level partly driven by positive Darwinian selection. We speculate that it is unlikely that the observed convergence at the nucleotide level has resulted in convergence at the functional level. Importantly, our molecular evolution analyses work suggest novel research questions regarding the “functional homology” between the New World monkeys RLN and the RLN1 and RLN2 genes from apes, and of the putative functional role of the C peptide, and the prorelaxin (i.e., the relaxin molecule that includes the C peptide).

## Supplementary Material

Supplementary table S1 is available at *Genome Biology and Evolution* online (http://www.gbe.oxfordjournals.org/).

Supplementary Data

## References

[evu023-B1] Arroyo JI, Hoffmann FG, Good S, Opazo JC (2012). INSL4 pseudogenes help define the relaxin family repertoire in the common ancestor of placental mammals. J Mol Evol..

[evu023-B2] Arroyo JI, Hoffmann FG, Opazo JC (2012a). Gene duplication and positive selection explains unusual physiological roles of the relaxin gene in the European rabbit. J Mol Evol..

[evu023-B3] Arroyo JI, Hoffmann FG, Opazo JC (2012b). Gene turnover and differential retention in the relaxin/insulin-like gene family in primates. Mol Phylogenet Evol..

[evu023-B5] Bathgate RA, Samuel CS, Burazin TC, Gundlach AL, Tregear GW (2003). Relaxin: new peptides, receptors and novel actions. Trends Endocrinol Metab..

[evu023-B4] Bathgate RA (2006). Relaxin-3: improved synthesis strategy and demonstration of its high-affinity interaction with the relaxin receptor LGR7 both in vitro and in vivo. Biochemistry.

[evu023-B6] Bieche I (2003). Placenta-specific INSL4 expression is mediated by a human endogenous retrovirus element. Biol Reprod..

[evu023-B7] Busse WD, Carpenter FH (1976). Synthesis and properties of carbonylbis(methionyl)insulin, a proinsulin analogue which is convertible to insulin by cyanogen bromide cleavage. Biochemistry.

[evu023-B8] Castoe TA (2009). Evidence for an ancient adaptive episode of convergent molecular evolution. Proc Natl Acad Sci U S A..

[evu023-B9] Diaz B, Pimentel B, de Pablo F, de La Rosa EJ (1999). Apoptotic cell death of proliferating neuroepithelial cells in the embryonic retina is prevented by insulin. Eur J Neurosci..

[evu023-B11] Einspanier A (1997). Local relaxin biosynthesis in the ovary and uterus through the oestrous cycle and early pregnancy in the female marmoset monkey (*Callithrix jacchus*). Hum Reprod..

[evu023-B10] Einspanier A (1999). Relaxin in the marmoset monkey: secretion pattern in the ovarian cycle and early pregnancy. Biol Reprod..

[evu023-B12] Eizinger A, Jungblut B, Sommer RJ (1999). Evolutionary change in the functional specificity of genes. Trends Genet..

[evu023-B13] Evans BA, Fu P, Tregear GW (1994). Characterization of two relaxin genes in the chimpanzee. J Endocrinol..

[evu023-B14] Hansell D, Bryant G, Greenwood F (1991). Expression of the human relaxin H1 gene in the decidua, trophoblast, and prostate. J Clin Endocrinol Metab..

[evu023-B15] Hernandez-Sanchez C, Mansilla A, de la Rosa EJ, de Pablo F (2006). Proinsulin in development: new roles for an ancient prohormone. Diabetologia.

[evu023-B16] Hernandez-Sanchez C, Rubio E, Serna J, de la Rosa EJ, de Pablo F (2002). Unprocessed proinsulin promotes cell survival during neurulation in the chick embryo. Diabetes.

[evu023-B17] Hoffmann FG, Opazo JC (2011). Evolution of the relaxin/insulin-like gene family in placental mammals: implications for its early evolution. J Mol Evol..

[evu023-B18] Hoffmann FG, Opazo JC, Storz JF (2010). Gene cooption and convergent evolution of oxygen transport hemoglobins in jawed and jawless vertebrates. Proc Natl Acad. Sci U S A..

[evu023-B19] Ivell R, Kotula-Balak M, Glynn D, Heng K, Anand-Ivell R (2011). Relaxin family peptides in the male reproductive system—a critical appraisal. Mol Hum Reprod..

[evu023-B20] Jobb G, Haeseler AV, Strimmer K (2004). TREEFINDER: a powerful graphical analysis environment for molecular phylogenetics. BMC Evol Biol..

[evu023-B21] Katoh K, Asimenos G, Toh H (2009). Multiple alignment of DNA sequences with MAFFT. Methods Mol Biol..

[evu023-B22] Li Y, Liu Z, Shi P, Zhang J (2010). The hearing gene Prestin unites echolocating bats and whales. Curr Biol..

[evu023-B23] Liu Y (2010). Convergent sequence evolution between echolocating bats and dolphins. Curr Biol..

[evu023-B24] Malaguarnera R (2012). Proinsulin binds with high affinity the insulin receptor isoform A and predominantly activates the mitogenic pathway. Endocrinology.

[evu023-B25] McGowan BM (2008). Relaxin-3 stimulates the hypothalamic-pituitary-gonadal axis. Am J Physiol Endocrinol Metab..

[evu023-B26] Nielsen R, Yang Z (1998). Likelihood models for detecting positively selected amino acid sites and applications to the HIV-1 envelope gene. Genetics.

[evu023-B27] Park JI, Chang CL, Hsu SY (2005). New Insights into biological roles of relaxin and relaxin-related peptides. Rev Endocr Metab Disord..

[evu023-B28] Park J-I, Semyonov J, Yi W, Chang CL, Hsu SYT (2008). Regulation of receptor signaling by relaxin A chain motifs: derivation of pan-specific and LGR7-specific human relaxin analogs. J Biol Chem..

[evu023-B29] Park J-I (2008). Origin of INSL3-mediated testicular descent in therian mammals. Genome Res..

[evu023-B30] Piatigorski J (2007). Gene sharing and evolution: the diversity of protein functions.

[evu023-B31] Ronquist F, Huelsenbeck JP (2003). MrBayes 3: Bayesian phylogenetic inference under mixed models. Bioinformatics.

[evu023-B32] Sakbun V, Ali SM, Greenwood FC, Bryant-Greenwood GD (1990). Human relaxin in the amnion, chorion, decidua parietalis, basal plate, and placental trophoblast by immunocytochemistry and northern analysis. J Clin Endocrinol Metab..

[evu023-B33] Sherwood OD (2004). Relaxin’s physiological roles and other diverse actions. Endocr Rev..

[evu023-B34] Shimodaira H, Hasegawa M (1999). Multiple comparisons of log-likelihoods with applications to phylogenetic inference. Mol Biol Evol..

[evu023-B35] Silvertown JD, Geddes BJ, Summerlee AJ (2003). Adenovirus-mediated expression of human prorelaxin promotes the invasive potential of canine mammary cancer cells. Endocrinology.

[evu023-B36] Tan YY, Wade JD, Tregear GW, Summers RJ (1998). Comparison of relaxin receptors in rat isolated atria and uterus by use of synthetic and native relaxin analogues. Br J Pharmacol..

[evu023-B37] Tatusova TA, Madden TL (1999). BLAST 2 Sequences, a new tool for comparing protein and nucleotide sequences. FEMS Microbiol Lett..

[evu023-B38] Vandlen R, Winslow J, Moffat B, Rinderknecht E, MacLennan AH, Tregear GW, Bryant-Greenwood GD (1995). Human relaxin: purification, characterization and production of recombinant relaxins for structure function studies. Progress in relaxin research: the proceedings of the second international congress on the hormone relaxin.

[evu023-B39] Wilkinson TN, Speed TP, Tregear GW, Bathgate RAD (2005). Evolution of the relaxin-like peptide family. BMC Evol Biol..

[evu023-B40] Yang Z (2007). PAML 4: phylogenetic analysis by maximum likelihood. Mol Biol Evol..

[evu023-B41] Yang Z, dos Reis M (2011). Statistical properties of the branch-site test of positive selection. Mol Biol Evol..

[evu023-B42] Yang Z, Nielsen R, Goldman N, Pedersen AM (2000). Codon-substitution models for heterogeneous selection pressure at amino acid sites. Genetics.

[evu023-B43] Yang Z, Wong WS, Nielsen R (2005). Bayes empirical Bayes inference of amino acid sites under positive selection. Mol Biol Evol..

[evu023-B44] Yegorov S, Good S (2012). Using paleogenomics to study the evolution of gene families: origin and duplication history of the relaxin family hormones and their receptors. PLoS One.

[evu023-B45] Yokoyama S, Altun A, DeNardo DF (2011). Molecular convergence of infrared vision in snakes. Mol Biol Evol..

[evu023-B46] Zarreh-Hoshyari-Khah R, Bartsch O, Einspanier A, Pohnke Y, Ivell R (2001). Bioactivity of recombinant prorelaxin from the marmoset monkey. Regul Pept..

[evu023-B47] Zhang J, Nielsen R, Yang Z (2005). Evaluation of an improved branch-site likelihood method for detecting positive selection at the molecular level. Mol Biol Evol..

