# Supplementary material for: Evolution of the Relaxin/Insulin-Like Gene Family in Anthropoid Primates
Source: Genome Biol Evol. 2014 Feb 2;6(3):491–9. doi: 10.1093/gbe/evu023 (PMC3971578; doi:10.1093/gbe/evu023)
Supplement: Supplementary Data [file supp_6_3_491__index.html]

Evolution of the Relaxin/Insulin-Like Gene Family in Anthropoid Primates — Supplementary Data 

# Evolution of the Relaxin/Insulin-Like Gene Family in Anthropoid Primates

## Supplementary Data

files

**Files in this Data Supplement:**

- Supplementary Data - pdf file
