# Supplementary material for: Evolution of the Relaxin/Insulin-Like Gene Family in Anthropoid Primates
Source: Genome Biol Evol. 2014 Feb 2;6(3):491–9. doi: 10.1093/gbe/evu023 (PMC3971578; doi:10.1093/gbe/evu023)
Supplement: Supplementary Data [file supp_evu023_Suppl_Table_S1.pdf]

Supplementary Table S1. List of accession numbers of DNA sequences used in comparative genomic analyses performed in this study.

Common name	Scientific name	Accession number
Human	*Homo sapiens*	Chr9:5,185,668-5,339,873
Chimpanzee	*Pan troglodytes*	Chr9:5,282,897-5,450,916
Bonobo	*Pan paniscus*	contig82418
Gorilla	*Gorilla gorilla*	Chr9:5,187,934-5,390,917
Bornean orangutan	*Pongo pygmaeus*	Chr9:57,547,568-57,803,411
Gibbon	*Nomascus leucogenys*	SuperContigGL397359.1:6,040,735-6,255,525
Rhesus monkey	*Macaca mulatta*	Chr15:71,839,443-72,032,294
Crab-eating monkey	*Macaca fascicularis*	contig167109
Hamadryas baboon	*Papio hamadryas*	Contig617816_Contig606745_Contig765783_Contig318249:49875-52817
Anubis baboon	*Papio anubis*	AHZZ01104622,AHZZ01104623,AHZZ01089265
Marmoset	*Callithrix jacchus*	Chr1:106,904,656-107,050,272
Squirrel monkey	*Saimiri boliviensis*	scaffold_JH378132.1:20000000:23605852
Philippine tarsier	*Tarsius syrichta*	ENSTSYG00000003808 (INSL6),ENSTSYG00000007147 (INSL4ps), ENSTSYG00000013076 (RLN2αps),ENSTSYG00000006242(RLN2βps), ENSTSYG00000001306(RLN2γps),ENSTSYG00000000722(RLN2δps),ENSTSYG00000006196(RLN2ε),ENSTSYG00000007074(RLN2ζps)
Bushbaby	*Otolemur garnettii*	ENSOGAG00000012255; ENSOGAG00000009500
Mouse lemur	*Microcebus murinus*	GeneScaffold_1014: 1,833-6,299
Malagasy lemur	*Varecia variegata rubra*	AF317424
African lorisiform	*Galago crassicaudatus*	AF317425
*Mouse	*Mus musculus*	Chr19: 29,395,837-29,409,160
*Rat	*Rattus norvegicus*	Chr1: 232,990,534-233,003,444
*Kangaroo rat	*Dipodomys ordii*	ENSDORG00000000108
*Dolphin	*Tursiops truncatus*	ENSTTRG00000011584
*Cattle	*Bos taurus*	ENSBTAG00000006651
*Pig	*Sus scrofa*	ENSSSCG00000005213
*Dog	*Canis familiaris*	Chr1: 96,525,702-96-96,570,855

NOTE.-* In order to attain a wide coverage of relaxin sequences, this species were included as outgroup in the phylogenetic context to calculate rates of substitution.
